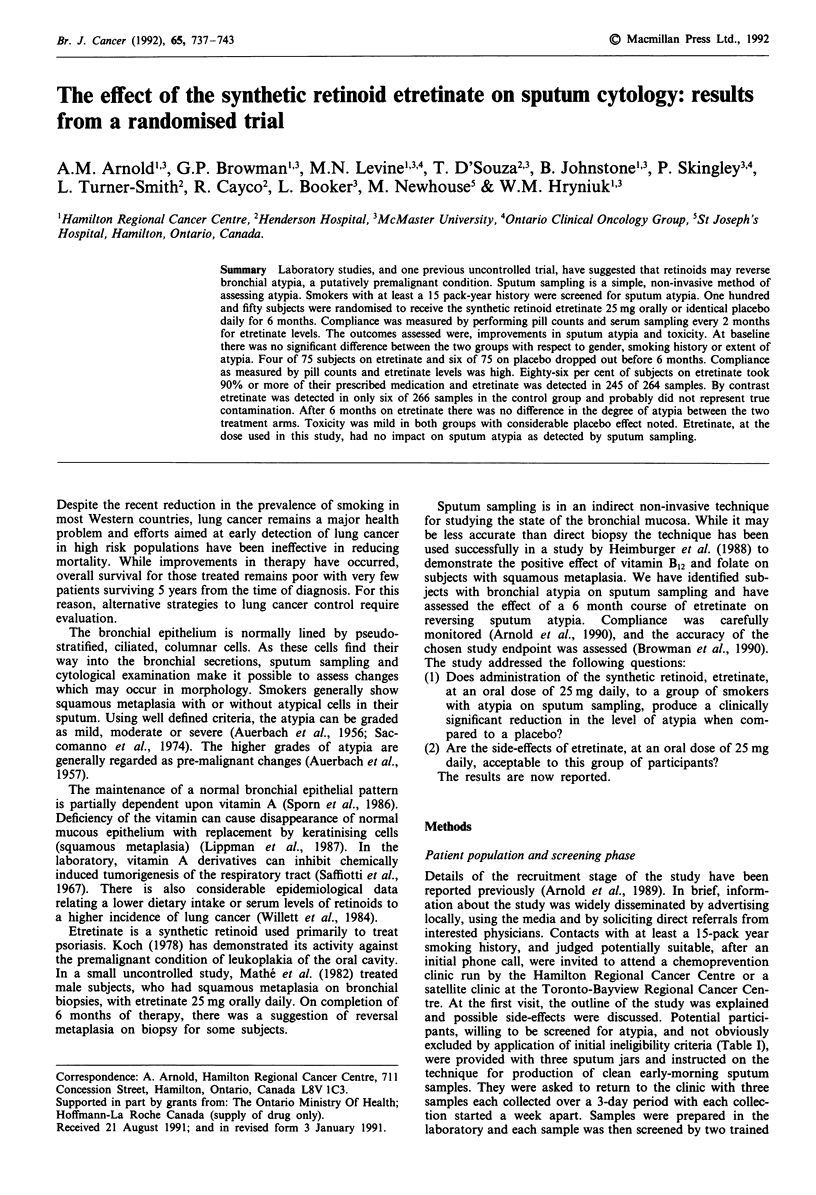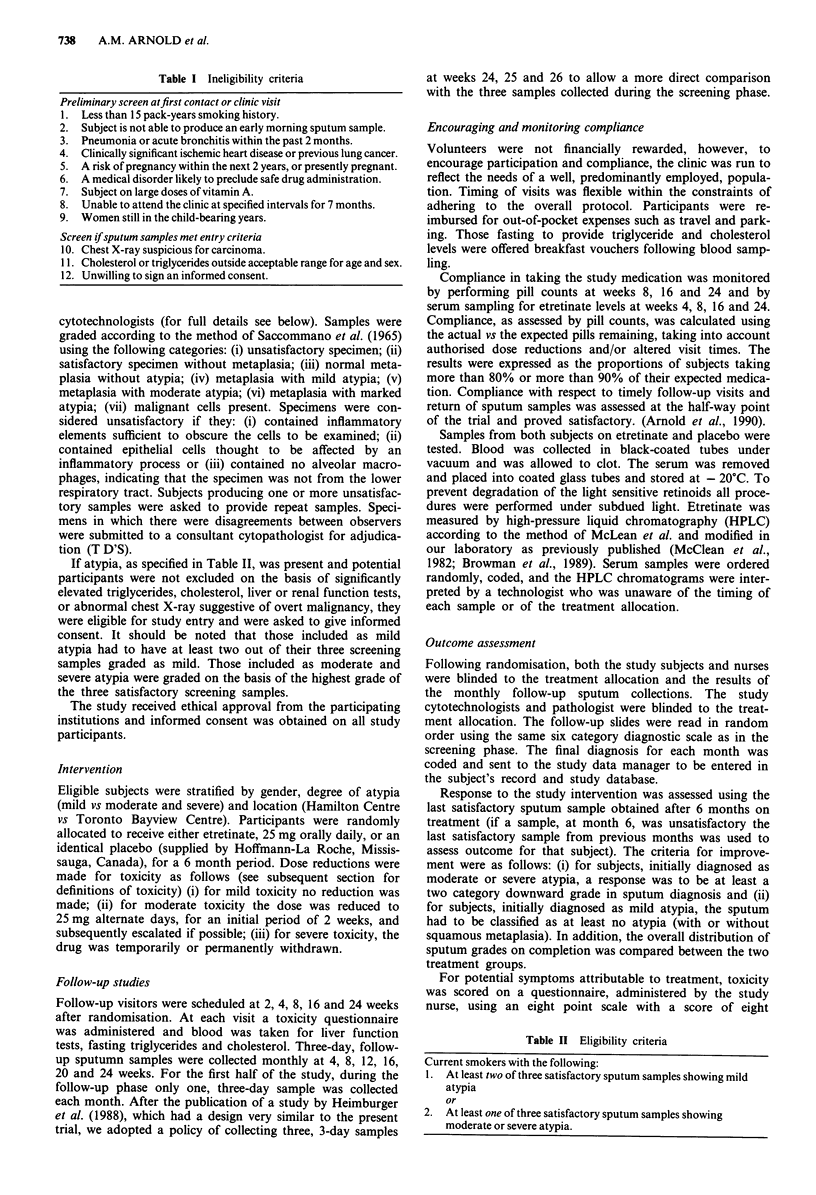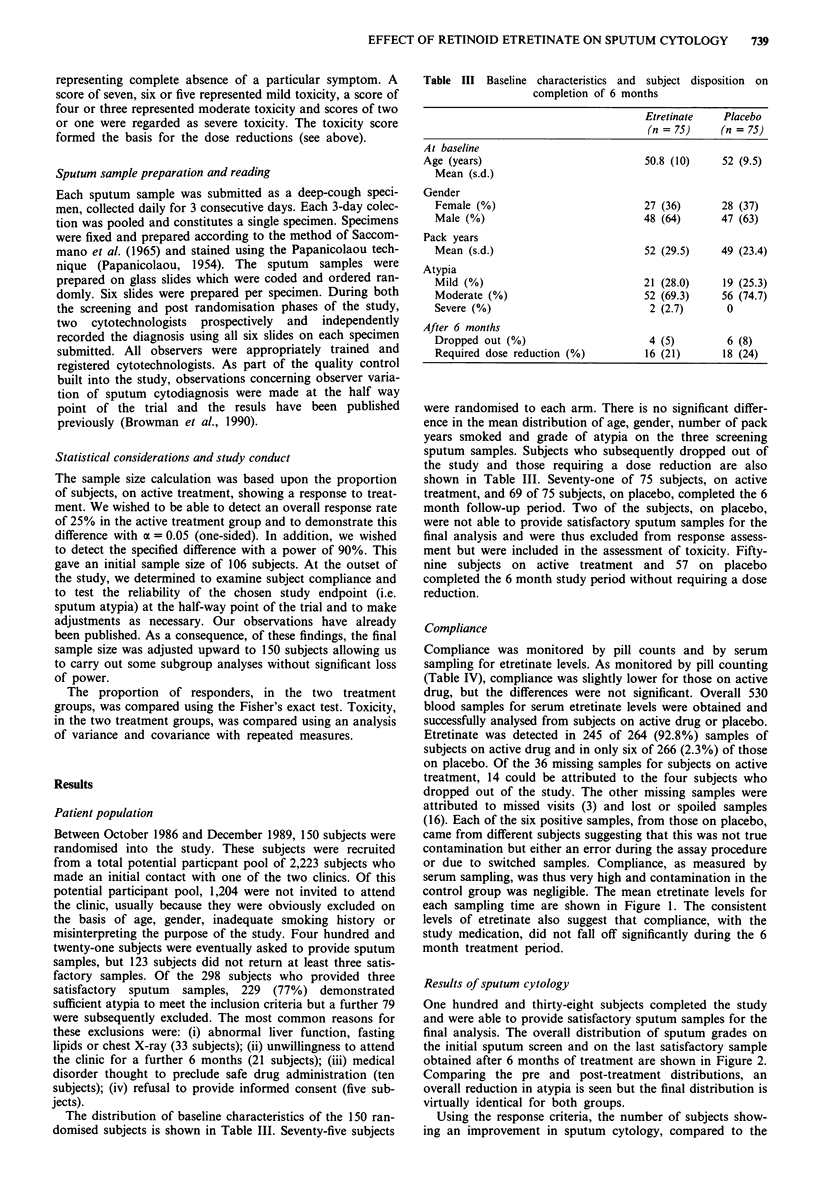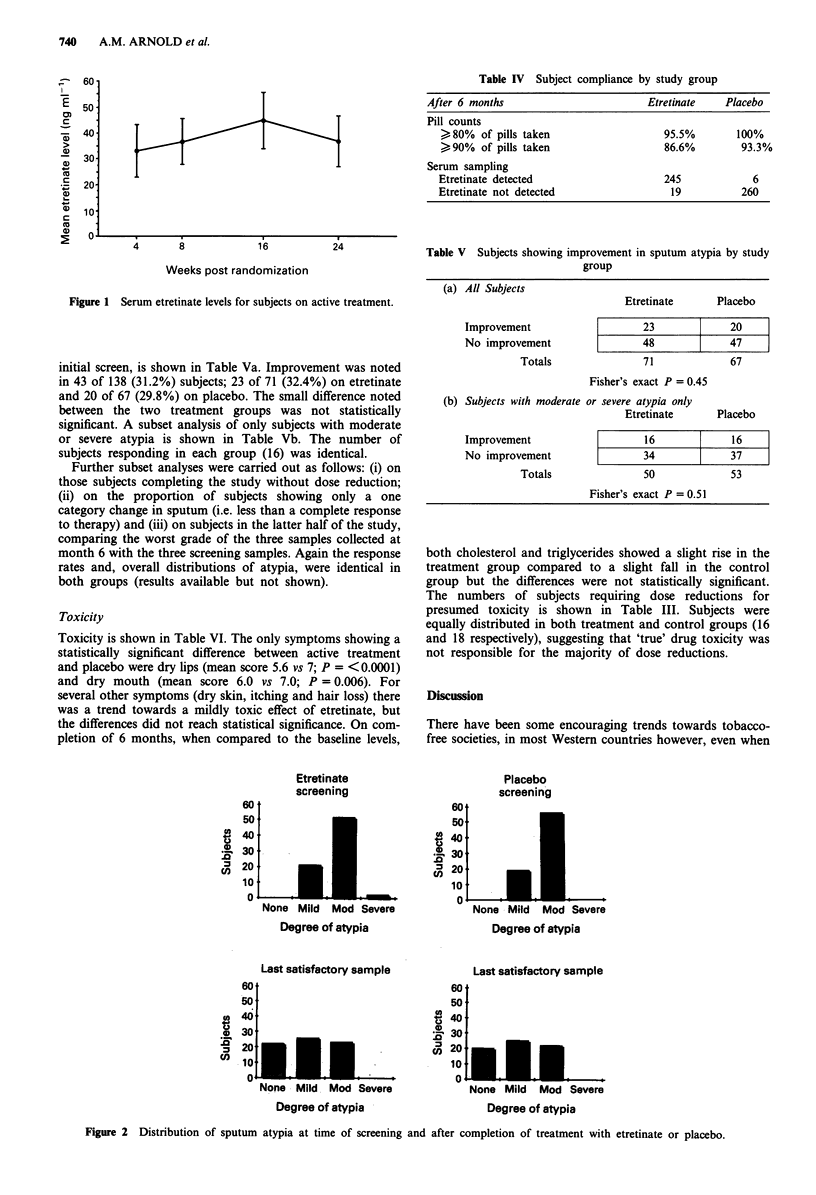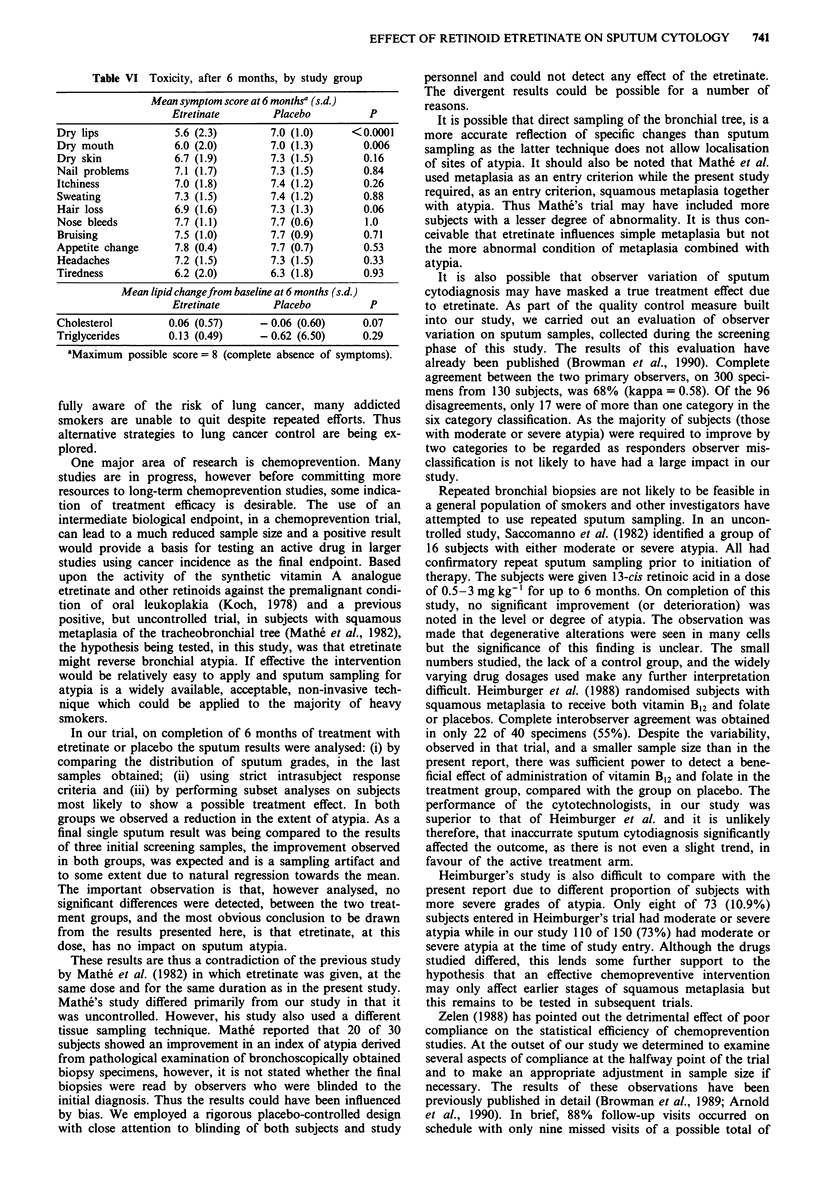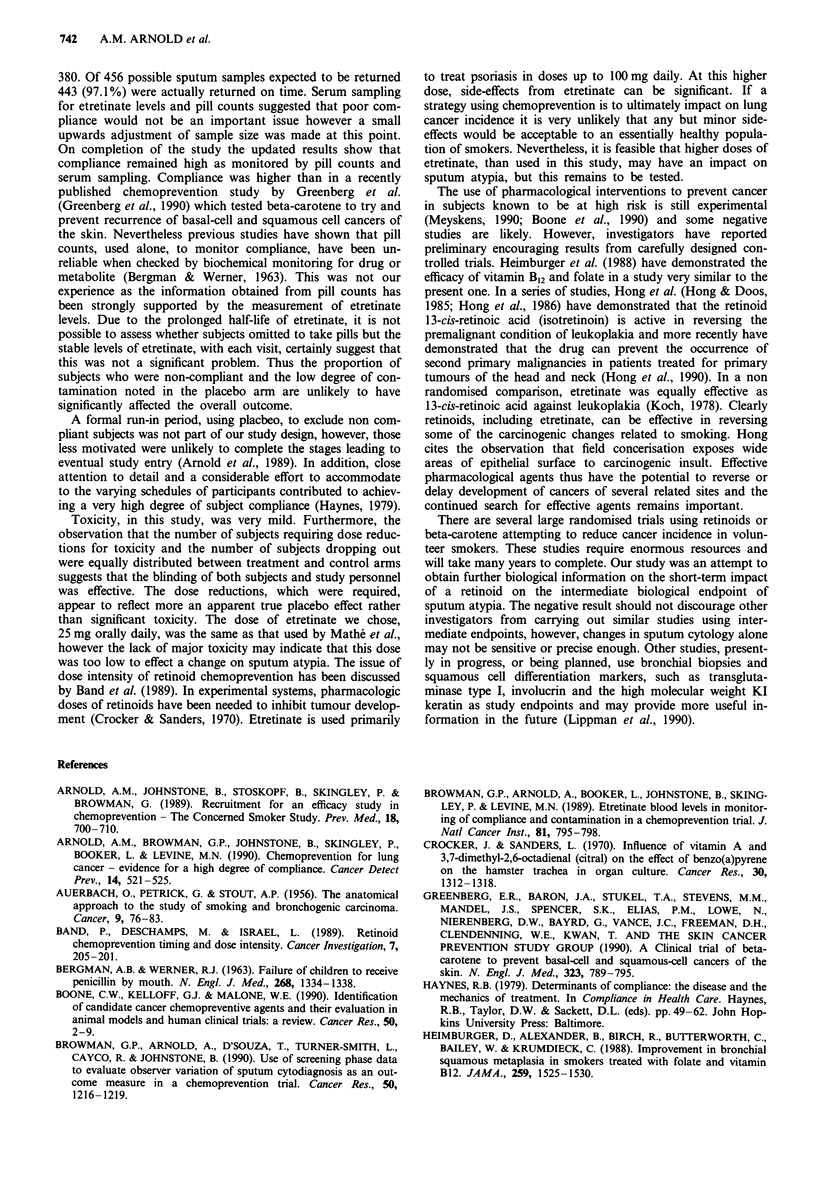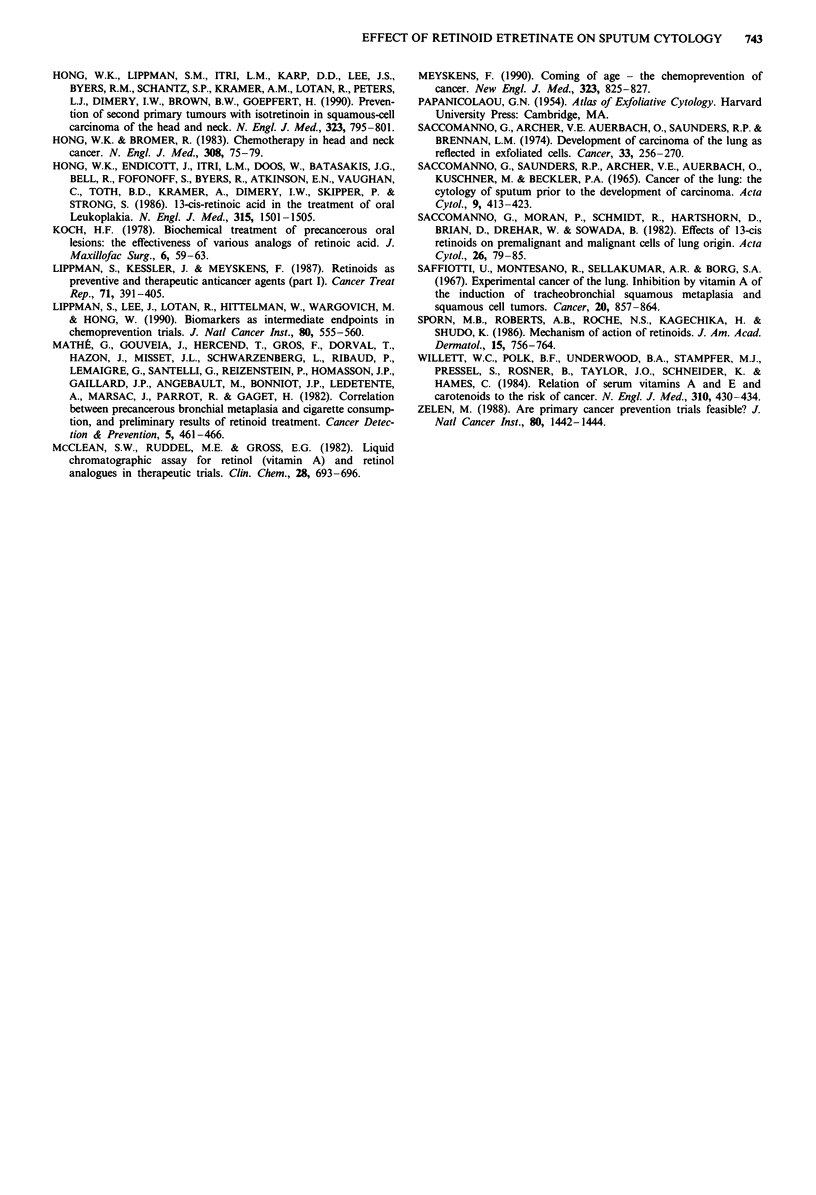# The effect of the synthetic retinoid etretinate on sputum cytology: results from a randomised trial.

**DOI:** 10.1038/bjc.1992.156

**Published:** 1992-05

**Authors:** A. M. Arnold, G. P. Browman, M. N. Levine, T. D'Souza, B. Johnstone, P. Skingley, L. Turner-Smith, R. Cayco, L. Booker, M. Newhouse

**Affiliations:** Hamilton Regional Cancer Centre, Ontario, Canada.

## Abstract

Laboratory studies, and one previous uncontrolled trial, have suggested that retinoids may reverse bronchial atypia, a putatively premalignant condition. Sputum sampling is a simple, non-invasive method of assessing atypia. Smokers with at least a 15 pack-year history were screened for sputum atypia. One hundred and fifty subjects' were randomised to receive the synthetic retinoid etretinate 25 mg orally or identical placebo daily for 6 months. Compliance was measured by performing pill counts and serum sampling every 2 months for etretinate levels. The outcomes assessed were, improvements in sputum atypia and toxicity. At baseline there was no significant difference between the two groups with respect to gender, smoking history or extent of atypia. Four of 75 subjects on etretinate and six of 75 on placebo dropped out before 6 months. Compliance as measured by pill counts and etretinate levels was high. Eighty-six per cent of subjects on etretinate took 90% or more of their prescribed medication and etretinate was detected in 245 of 264 samples. By contrast etretinate was detected in only six of 266 samples in the control group and probably did not represent true contamination. After 6 months on etretinate there was no difference in the degree of atypia between the two treatment arms. Toxicity was mild in both groups with considerable placebo effect noted. Etretinate, at the dose used in this study, had no impact on sputum atypia as detected by sputum sampling.


Br. J. Cancer (1992), 65, 737–743 © Macmillan Press Ltd., 1992

### The effect of the synthetic retinoid etretinate on sputum cytology: results from a randomised trial

A.M. Arnold[1,3], G.P. Browman[1,3], M.N. Levine[1,3,4], T. D'Souza[2,3], B. Johnstone[1,3], P. Skingley[3,4], L. Turner-Smith[2], R. Cayco[2], L. Booker[3], M. Newhouse[5] & W.M. Hryniuk[1,3]

[1]*Hamilton Regional Cancer Centre,* [2]*Henderson Hospital,* [3]*McMaster University,* [4]*Ontario Clinical Oncology Group,* [5]*St Joseph's Hospital, Hamilton, Ontario, Canada.*

**Summary** Laboratory studies, and one previous uncontrolled trial, have suggested that retinoids may reverse bronchial atypia, a putatively premalignant condition. Sputum sampling is a simple, non-invasive method of assessing atypia. Smokers with at least a 15 pack-year history were screened for sputum atypia. One hundred and fifty subjects were randomised to receive the synthetic retinoid etretinate 25 mg orally or identical placebo daily for 6 months. Compliance was measured by performing pill counts and serum sampling every 2 months for etretinate levels. The outcomes assessed were, improvements in sputum atypia and toxicity. At baseline there was no significant difference between the two groups with respect to gender, smoking history or extent of atypia. Four of 75 subjects on etretinate and six of 75 on placebo dropped out before 6 months. Compliance as measured by pill counts and etretinate levels was high. Eighty-six per cent of subjects on etretinate took 90% or more of their prescribed medication and etretinate was detected in 245 of 264 samples. By contrast etretinate was detected in only six of 266 samples in the control group and probably did not represent true contamination. After 6 months on etretinate there was no difference in the degree of atypia between the two treatment arms. Toxicity was mild in both groups with considerable placebo effect noted. Etretinate, at the dose used in this study, had no impact on sputum atypia as detected by sputum sampling.

Despite the recent reduction in the prevalence of smoking in most Western countries, lung cancer remains a major health problem and efforts aimed at early detection of lung cancer in high risk populations have been ineffective in reducing mortality. While improvements in therapy have occurred, overall survival for those treated remains poor with very few patients surviving 5 years from the time of diagnosis. For this reason, alternative strategies to lung cancer control require evaluation.

The bronchial epithelium is normally lined by pseudo-stratified, ciliated, columnar cells. As these cells find their way into the bronchial secretions, sputum sampling and cytological examination make it possible to assess changes which may occur in morphology. Smokers generally show squamous metaplasia with or without atypical cells in their sputum. Using well defined criteria, the atypia can be graded as mild, moderate or severe (Auerbach *et al.*, 1956; Saccomanno *et al.*, 1974). The higher grades of atypia are generally regarded as pre-malignant changes (Auerbach *et al.*, 1957).

The maintenance of a normal bronchial epithelial pattern is partially dependent upon vitamin A (Sporn *et al.*, 1986). Deficiency of the vitamin can cause disappearance of normal mucous epithelium with replacement by keratinising cells (squamous metaplasia) (Lippman *et al.*, 1987). In the laboratory, vitamin A derivatives can inhibit chemically induced tumorigenesis of the respiratory tract (Saffiotti *et al.*, 1967). There is also considerable epidemiological data relating a lower dietary intake or serum levels of retinoids to a higher incidence of lung cancer (Willett *et al.*, 1984).

Etretinate is a synthetic retinoid used primarily to treat psoriasis. Koch (1978) has demonstrated its activity against the premalignant condition of leukoplakia of the oral cavity. In a small uncontrolled study, Mathé *et al.* (1982) treated male subjects, who had squamous metaplasia on bronchial biopsies, with etretinate 25 mg orally daily. On completion of 6 months of therapy, there was a suggestion of reversal metaplasia on biopsy for some subjects.

Correspondence: A. Arnold, Hamilton Regional Cancer Centre, 711 Concession Street, Hamilton, Ontario, Canada L8V 1C3.
Supported in part by grants from: The Ontario Ministry Of Health; Hoffmann-La Roche Canada (supply of drug only).
Received 21 August 1991; and in revised form 3 January 1991.

Sputum sampling is in an indirect non-invasive technique for studying the state of the bronchial mucosa. While it may be less accurate than direct biopsy the technique has been used successfully in a study by Heimburger *et al.* (1988) to demonstrate the positive effect of vitamin $B_{12}$ and folate on subjects with squamous metaplasia. We have identified subjects with bronchial atypia on sputum sampling and have assessed the effect of a 6 month course of etretinate on reversing sputum atypia. Compliance was carefully monitored (Arnold *et al.*, 1990), and the accuracy of the chosen study endpoint was assessed (Browman *et al.*, 1990). The study addressed the following questions:

(1) Does administration of the synthetic retinoid, etretinate, at an oral dose of 25 mg daily, to a group of smokers with atypia on sputum sampling, produce a clinically significant reduction in the level of atypia when compared to a placebo?
(2) Are the side-effects of etretinate, at an oral dose of 25 mg daily, acceptable to this group of participants?

The results are now reported.

### Methods

### *Patient population and screening phase*

Details of the recruitment stage of the study have been reported previously (Arnold *et al.*, 1989). In brief, information about the study was widely disseminated by advertising locally, using the media and by soliciting direct referrals from interested physicians. Contacts with at least a 15-pack year smoking history, and judged potentially suitable, after an initial phone call, were invited to attend a chemoprevention clinic run by the Hamilton Regional Cancer Centre or a satellite clinic at the Toronto-Bayview Regional Cancer Centre. At the first visit, the outline of the study was explained and possible side-effects were discussed. Potential participants, willing to be screened for atypia, and not obviously excluded by application of initial ineligibility criteria (Table I), were provided with three sputum jars and instructed on the technique for production of clean early-morning sputum samples. They were asked to return to the clinic with three samples each collected over a 3-day period with each collection started a week apart. Samples were prepared in the laboratory and each sample was then screened by two trained

Table I Ineligibility criteria

| |
|---|
| *Preliminary screen at first contact or clinic visit* |
| 1. Less than 15 pack-years smoking history. |
| 2. Subject is not able to produce an early morning sputum sample. |
| 3. Pneumonia or acute bronchitis within the past 2 months. |
| 4. Clinically significant ischemic heart disease or previous lung cancer. |
| 5. A risk of pregnancy within the next 2 years, or presently pregnant. |
| 6. A medical disorder likely to preclude safe drug administration. |
| 7. Subject on large doses of vitamin A. |
| 8. Unable to attend the clinic at specified intervals for 7 months. |
| 9. Women still in the child-bearing years. |
| *Screen if sputum samples met entry criteria* |
| 10. Chest X-ray suspicious for carcinoma. |
| 11. Cholesterol or triglycerides outside acceptable range for age and sex. |
| 12. Unwilling to sign an informed consent. |

cytotechnologists (for full details see below). Samples were graded according to the method of Saccommano *et al.* (1965) using the following categories: (i) unsatisfactory specimen; (ii) satisfactory specimen without metaplasia; (iii) normal metaplasia without atypia; (iv) metaplasia with mild atypia; (v) metaplasia with moderate atypia; (vi) metaplasia with marked atypia; (vii) malignant cells present. Specimens were considered unsatisfactory if they: (i) contained inflammatory elements sufficient to obscure the cells to be examined; (ii) contained epithelial cells thought to be affected by an inflammatory process or (iii) contained no alveolar macrophages, indicating that the specimen was not from the lower respiratory tract. Subjects producing one or more unsatisfactory samples were asked to provide repeat samples. Specimens in which there were disagreements between observers were submitted to a consultant cytopathologist for adjudication (T D'S).

If atypia, as specified in Table II, was present and potential participants were not excluded on the basis of significantly elevated triglycerides, cholesterol, liver or renal function tests, or abnormal chest X-ray suggestive of overt malignancy, they were eligible for study entry and were asked to give informed consent. It should be noted that those included as mild atypia had to have at least two out of their three screening samples graded as mild. Those included as moderate and severe atypia were graded on the basis of the highest grade of the three satisfactory screening samples.

The study received ethical approval from the participating institutions and informed consent was obtained on all study participants.

### *Intervention*

Eligible subjects were stratified by gender, degree of atypia (mild *vs* moderate and severe) and location (Hamilton Centre *vs* Toronto Bayview Centre). Participants were randomly allocated to receive either etretinate, 25 mg orally daily, or an identical placebo (supplied by Hoffmann-La Roche, Mississauga, Canada), for a 6 month period. Dose reductions were made for toxicity as follows (see subsequent section for definitions of toxicity) (i) for mild toxicity no reduction was made; (ii) for moderate toxicity the dose was reduced to 25 mg alternate days, for an initial period of 2 weeks, and subsequently escalated if possible; (iii) for severe toxicity, the drug was temporarily or permanently withdrawn.

### *Follow-up studies*

Follow-up visitors were scheduled at 2, 4, 8, 16 and 24 weeks after randomisation. At each visit a toxicity questionnaire was administered and blood was taken for liver function tests, fasting triglycerides and cholesterol. Three-day, follow-up sputumn samples were collected monthly at 4, 8, 12, 16, 20 and 24 weeks. For the first half of the study, during the follow-up phase only one, three-day sample was collected each month. After the publication of a study by Heimburger *et al.* (1988), which had a design very similar to the present trial, we adopted a policy of collecting three, 3-day samples at weeks 24, 25 and 26 to allow a more direct comparison with the three samples collected during the screening phase.

### *Encouraging and monitoring compliance*

Volunteers were not financially rewarded, however, to encourage participation and compliance, the clinic was run to reflect the needs of a well, predominantly employed, population. Timing of visits was flexible within the constraints of adhering to the overall protocol. Participants were reimbursed for out-of-pocket expenses such as travel and parking. Those fasting to provide triglyceride and cholesterol levels were offered breakfast vouchers following blood sampling.

Compliance in taking the study medication was monitored by performing pill counts at weeks 8, 16 and 24 and by serum sampling for etretinate levels at weeks 4, 8, 16 and 24. Compliance, as assessed by pill counts, was calculated using the actual *vs* the expected pills remaining, taking into account authorised dose reductions and/or altered visit times. The results were expressed as the proportions of subjects taking more than 80% or more than 90% of their expected medication. Compliance with respect to timely follow-up visits and return of sputum samples was assessed at the half-way point of the trial and proved satisfactory. (Arnold *et al.*, 1990).

Samples from both subjects on etretinate and placebo were tested. Blood was collected in black-coated tubes under vacuum and was allowed to clot. The serum was removed and placed into coated glass tubes and stored at − 20°C. To prevent degradation of the light sensitive retinoids all procedures were performed under subdued light. Etretinate was measured by high-pressure liquid chromatography (HPLC) according to the method of McLean *et al.* and modified in our laboratory as previously published (McClean *et al.*, 1982; Browman *et al.*, 1989). Serum samples were ordered randomly, coded, and the HPLC chromatograms were interpreted by a technologist who was unaware of the timing of each sample or of the treatment allocation.

### *Outcome assessment*

Following randomisation, both the study subjects and nurses were blinded to the treatment allocation and the results of the monthly follow-up sputum collections. The study cytotechnologists and pathologist were blinded to the treatment allocation. The follow-up slides were read in random order using the same six category diagnostic scale as in the screening phase. The final diagnosis for each month was coded and sent to the study data manager to be entered in the subject's record and study database.

Response to the study intervention was assessed using the last satisfactory sputum sample obtained after 6 months on treatment (if a sample, at month 6, was unsatisfactory the last satisfactory sample from previous months was used to assess outcome for that subject). The criteria for improvement were as follows: (i) for subjects, initially diagnosed as moderate or severe atypia, a response was to be at least a two category downward grade in sputum diagnosis and (ii) for subjects, initially diagnosed as mild atypia, the sputum had to be classified as at least no atypia (with or without squamous metaplasia). In addition, the overall distribution of sputum grades on completion was compared between the two treatment groups.

For potential symptoms attributable to treatment, toxicity was scored on a questionnaire, administered by the study nurse, using an eight point scale with a score of eight

Table II Eligibility criteria

| |
|---|
| Current smokers with the following: |
| 1. At least *two* of three satisfactory sputum samples showing mild atypia |
| *or* |
| 2. At least *one* of three satisfactory sputum samples showing moderate or severe atypia. |

representing complete absence of a particular symptom. A score of seven, six or five represented mild toxicity, a score of four or three represented moderate toxicity and scores of two or one were regarded as severe toxicity. The toxicity score formed the basis for the dose reductions (see above).

*Sputum sample preparation and reading*

Each sputum sample was submitted as a deep-cough specimen, collected daily for 3 consecutive days. Each 3-day colection was pooled and constitutes a single specimen. Specimens were fixed and prepared according to the method of Saccommano *et al.* (1965) and stained using the Papanicolaou technique (Papanicolaou, 1954). The sputum samples were prepared on glass slides which were coded and ordered randomly. Six slides were prepared per specimen. During both the screening and post randomisation phases of the study, two cytotechnologists prospectively and independently recorded the diagnosis using all six slides on each specimen submitted. All observers were appropriately trained and registered cytotechnologists. As part of the quality control built into the study, observations concerning observer variation of sputum cytodiagnosis were made at the half way point of the trial and the resuls have been published previously (Browman *et al.*, 1990).

*Statistical considerations and study conduct*

The sample size calculation was based upon the proportion of subjects, on active treatment, showing a response to treatment. We wished to be able to detect an overall response rate of 25% in the active treatment group and to demonstrate this difference with $\alpha = 0.05$ (one-sided). In addition, we wished to detect the specified difference with a power of 90%. This gave an initial sample size of 106 subjects. At the outset of the study, we determined to examine subject compliance and to test the reliability of the chosen study endpoint (i.e. sputum atypia) at the half-way point of the trial and to make adjustments as necessary. Our observations have already been published. As a consequence, of these findings, the final sample size was adjusted upward to 150 subjects allowing us to carry out some subgroup analyses without significant loss of power.

The proportion of responders, in the two treatment groups, was compared using the Fisher's exact test. Toxicity, in the two treatment groups, was compared using an analysis of variance and covariance with repeated measures.

### Results

*Patient population*

Between October 1986 and December 1989, 150 subjects were randomised into the study. These subjects were recruited from a total potential particpant pool of 2,223 subjects who made an initial contact with one of the two clinics. Of this potential participant pool, 1,204 were not invited to attend the clinic, usually because they were obviously excluded on the basis of age, gender, inadequate smoking history or misinterpreting the purpose of the study. Four hundred and twenty-one subjects were eventually asked to provide sputum samples, but 123 subjects did not return at least three satisfactory samples. Of the 298 subjects who provided three satisfactory sputum samples, 229 (77%) demonstrated sufficient atypia to meet the inclusion criteria but a further 79 were subsequently excluded. The most common reasons for these exclusions were: (i) abnormal liver function, fasting lipids or chest X-ray (33 subjects); (ii) unwillingness to attend the clinic for a further 6 months (21 subjects); (iii) medical disorder thought to preclude safe drug administration (ten subjects); (iv) refusal to provide informed consent (five subjects).

The distribution of baseline characteristics of the 150 randomised subjects is shown in Table III. Seventy-five subjects

**Table III** Baseline characteristics and subject disposition on completion of 6 months

| | *Etretinate (n = 75)* | *Placebo (n = 75)* |
|---|---|---|
| *At baseline* | | |
| Age (years) Mean (s.d.) | 50.8 (10) | 52 (9.5) |
| Gender | | |
| Female (%) | 27 (36) | 28 (37) |
| Male (%) | 48 (64) | 47 (63) |
| Pack years | | |
| Mean (s.d.) | 52 (29.5) | 49 (23.4) |
| Atypia | | |
| Mild (%) | 21 (28.0) | 19 (25.3) |
| Moderate (%) | 52 (69.3) | 56 (74.7) |
| Severe (%) | 2 (2.7) | 0 |
| *After 6 months* | | |
| Dropped out (%) | 4 (5) | 6 (8) |
| Required dose reduction (%) | 16 (21) | 18 (24) |

were randomised to each arm. There is no significant difference in the mean distribution of age, gender, number of pack years smoked and grade of atypia on the three screening sputum samples. Subjects who subsequently dropped out of the study and those requiring a dose reduction are also shown in Table III. Seventy-one of 75 subjects, on active treatment, and 69 of 75 subjects, on placebo, completed the 6 month follow-up period. Two of the subjects, on placebo, were not able to provide satisfactory sputum samples for the final analysis and were thus excluded from response assessment but were included in the assessment of toxicity. Fifty-nine subjects on active treatment and 57 on placebo completed the 6 month study period without requiring a dose reduction.

*Compliance*

Compliance was monitored by pill counts and by serum sampling for etretinate levels. As monitored by pill counting (Table IV), compliance was slightly lower for those on active drug, but the differences were not significant. Overall 530 blood samples for serum etretinate levels were obtained and successfully analysed from subjects on active drug or placebo. Etretinate was detected in 245 of 264 (92.8%) samples of subjects on active drug and in only six of 266 (2.3%) of those on placebo. Of the 36 missing samples for subjects on active treatment, 14 could be attributed to the four subjects who dropped out of the study. The other missing samples were attributed to missed visits (3) and lost or spoiled samples (16). Each of the six positive samples, from those on placebo, came from different subjects suggesting that this was not true contamination but either an error during the assay procedure or due to switched samples. Compliance, as measured by serum sampling, was thus very high and contamination in the control group was negligible. The mean etretinate levels for each sampling time are shown in Figure 1. The consistent levels of etretinate also suggest that compliance, with the study medication, did not fall off significantly during the 6 month treatment period.

*Results of sputum cytology*

One hundred and thirty-eight subjects completed the study and were able to provide satisfactory sputum samples for the final analysis. The overall distribution of sputum grades on the initial sputum screen and on the last satisfactory sample obtained after 6 months of treatment are shown in Figure 2. Comparing the pre and post-treatment distributions, an overall reduction in atypia is seen but the final distribution is virtually identical for both groups.

Using the response criteria, the number of subjects showing an improvement in sputum cytology, compared to the

**Figure 1** Serum etretinate levels for subjects on active treatment.

initial screen, is shown in Table Va. Improvement was noted in 43 of 138 (31.2%) subjects; 23 of 71 (32.4%) on etretinate and 20 of 67 (29.8%) on placebo. The small difference noted between the two treatment groups was not statistically significant. A subset analysis of only subjects with moderate or severe atypia is shown in Table Vb. The number of subjects responding in each group (16) was identical.

Further subset analyses were carried out as follows: (i) on those subjects completing the study without dose reduction; (ii) on the proportion of subjects showing only a one category change in sputum (i.e. less than a complete response to therapy) and (iii) on subjects in the latter half of the study, comparing the worst grade of the three samples collected at month 6 with the three screening samples. Again the response rates and, overall distributions of atypia, were identical in both groups (results available but not shown).

### *Toxicity*

Toxicity is shown in Table VI. The only symptoms showing a statistically significant difference between active treatment and placebo were dry lips (mean score 5.6 *vs* 7; $P = <0.0001$) and dry mouth (mean score 6.0 *vs* 7.0; $P = 0.006$). For several other symptoms (dry skin, itching and hair loss) there was a trend towards a mildly toxic effect of etretinate, but the differences did not reach statistical significance. On completion of 6 months, when compared to the baseline levels,

**Table IV** Subject compliance by study group

| *After 6 months* | *Etretinate* | *Placebo* |
|---|---|---|
| Pill counts | | |
| ⩾80% of pills taken | 95.5% | 100% |
| ⩾90% of pills taken | 86.6% | 93.3% |
| Serum sampling | | |
| Etretinate detected | 245 | 6 |
| Etretinate not detected | 19 | 260 |

**Table V** Subjects showing improvement in sputum atypia by study group

(a) *All Subjects*

| | Etretinate | Placebo |
|---|---|---|
| Improvement | 23 | 20 |
| No improvement | 48 | 47 |
| Totals | 71 | 67 |

Fisher's exact $P = 0.45$

(b) *Subjects with moderate or severe atypia only*

| | Etretinate | Placebo |
|---|---|---|
| Improvement | 16 | 16 |
| No improvement | 34 | 37 |
| Totals | 50 | 53 |

Fisher's exact $P = 0.51$

both cholesterol and triglycerides showed a slight rise in the treatment group compared to a slight fall in the control group but the differences were not statistically significant. The numbers of subjects requiring dose reductions for presumed toxicity is shown in Table III. Subjects were equally distributed in both treatment and control groups (16 and 18 respectively), suggesting that 'true' drug toxicity was not responsible for the majority of dose reductions.

### Discussion

There have been some encouraging trends towards tobacco-free societies, in most Western countries however, even when

**Figure 2** Distribution of sputum atypia at time of screening and after completion of treatment with etretinate or placebo.

**Table VI** Toxicity, after 6 months, by study group

| | *Mean symptom score at 6 months*[a] *(s.d.)* | | |
|---|---|---|---|
| | *Etretinate* | *Placebo* | *P* |
| Dry lips | 5.6 (2.3) | 7.0 (1.0) | $<0.0001$ |
| Dry mouth | 6.0 (2.0) | 7.0 (1.3) | 0.006 |
| Dry skin | 6.7 (1.9) | 7.3 (1.5) | 0.16 |
| Nail problems | 7.1 (1.7) | 7.3 (1.5) | 0.84 |
| Itchiness | 7.0 (1.8) | 7.4 (1.2) | 0.26 |
| Sweating | 7.3 (1.5) | 7.4 (1.2) | 0.88 |
| Hair loss | 6.9 (1.6) | 7.3 (1.3) | 0.06 |
| Nose bleeds | 7.7 (1.1) | 7.7 (0.6) | 1.0 |
| Bruising | 7.5 (1.0) | 7.7 (0.9) | 0.71 |
| Appetite change | 7.8 (0.4) | 7.7 (0.7) | 0.53 |
| Headaches | 7.2 (1.5) | 7.3 (1.5) | 0.33 |
| Tiredness | 6.2 (2.0) | 6.3 (1.8) | 0.93 |
| | *Mean lipid change from baseline at 6 months (s.d.)* | | |
| | *Etretinate* | *Placebo* | *P* |
| Cholesterol | 0.06 (0.57) | − 0.06 (0.60) | 0.07 |
| Triglycerides | 0.13 (0.49) | − 0.62 (6.50) | 0.29 |

[a]Maximum possible score = 8 (complete absence of symptoms).

fully aware of the risk of lung cancer, many addicted smokers are unable to quit despite repeated efforts. Thus alternative strategies to lung cancer control are being explored.

One major area of research is chemoprevention. Many studies are in progress, however before committing more resources to long-term chemoprevention studies, some indication of treatment efficacy is desirable. The use of an intermediate biological endpoint, in a chemoprevention trial, can lead to a much reduced sample size and a positive result would provide a basis for testing an active drug in larger studies using cancer incidence as the final endpoint. Based upon the activity of the synthetic vitamin A analogue etretinate and other retinoids against the premalignant condition of oral leukoplakia (Koch, 1978) and a previous positive, but uncontrolled trial, in subjects with squamous metaplasia of the tracheobronchial tree (Mathé *et al.*, 1982), the hypothesis being tested, in this study, was that etretinate might reverse bronchial atypia. If effective the intervention would be relatively easy to apply and sputum sampling for atypia is a widely available, acceptable, non-invasive technique which could be applied to the majority of heavy smokers.

In our trial, on completion of 6 months of treatment with etretinate or placebo the sputum results were analysed: (i) by comparing the distribution of sputum grades, in the last samples obtained; (ii) using strict intrasubject response criteria and (iii) by performing subset analyses on subjects most likely to show a possible treatment effect. In both groups we observed a reduction in the extent of atypia. As a final single sputum result was being compared to the results of three initial screening samples, the improvement observed in both groups, was expected and is a sampling artifact and to some extent due to natural regression towards the mean. The important observation is that, however analysed, no significant differences were detected, between the two treatment groups, and the most obvious conclusion to be drawn from the results presented here, is that etretinate, at this dose, has no impact on sputum atypia.

These results are thus a contradiction of the previous study by Mathé *et al.* (1982) in which etretinate was given, at the same dose and for the same duration as in the present study. Mathé's study differed primarily from our study in that it was uncontrolled. However, his study also used a different tissue sampling technique. Mathé reported that 20 of 30 subjects showed an improvement in an index of atypia derived from pathological examination of bronchoscopically obtained biopsy specimens, however, it is not stated whether the final biopsies were read by observers who were blinded to the initial diagnosis. Thus the results could have been influenced by bias. We employed a rigorous placebo-controlled design with close attention to blinding of both subjects and study personnel and could not detect any effect of the etretinate. The divergent results could be possible for a number of reasons.

It is possible that direct sampling of the bronchial tree, is a more accurate reflection of specific changes than sputum sampling as the latter technique does not allow localisation of sites of atypia. It should also be noted that Mathé *et al.* used metaplasia as an entry criterion while the present study required, as an entry criterion, squamous metaplasia together with atypia. Thus Mathé's trial may have included more subjects with a lesser degree of abnormality. It is thus conceivable that etretinate influences simple metaplasia but not the more abnormal condition of metaplasia combined with atypia.

It is also possible that observer variation of sputum cytodiagnosis may have masked a true treatment effect due to etretinate. As part of the quality control measure built into our study, we carried out an evaluation of observer variation on sputum samples, collected during the screening phase of this study. The results of this evaluation have already been published (Browman *et al.*, 1990). Complete agreement between the two primary observers, on 300 specimens from 130 subjects, was 68% (kappa = 0.58). Of the 96 disagreements, only 17 were of more than one category in the six category classification. As the majority of subjects (those with moderate or severe atypia) were required to improve by two categories to be regarded as responders observer misclassification is not likely to have had a large impact in our study.

Repeated bronchial biopsies are not likely to be feasible in a general population of smokers and other investigators have attempted to use repeated sputum sampling. In an uncontrolled study, Saccomanno *et al.* (1982) identified a group of 16 subjects with either moderate or severe atypia. All had confirmatory repeat sputum sampling prior to initiation of therapy. The subjects were given 13-*cis* retinoic acid in a dose of $0.5-3\ mg\ kg^{-1}$ for up to 6 months. On completion of this study, no significant improvement (or deterioration) was noted in the level or degree of atypia. The observation was made that degenerative alterations were seen in many cells but the significance of this finding is unclear. The small numbers studied, the lack of a control group, and the widely varying drug dosages used make any further interpretation difficult. Heimburger *et al.* (1988) randomised subjects with squamous metaplasia to receive both vitamin $B_{12}$ and folate or placebos. Complete interobserver agreement was obtained in only 22 of 40 specimens (55%). Despite the variability, observed in that trial, and a smaller sample size than in the present report, there was sufficient power to detect a beneficial effect of administration of vitamin $B_{12}$ and folate in the treatment group, compared with the group on placebo. The performance of the cytotechnologists, in our study was superior to that of Heimburger *et al.* and it is unlikely therefore, that inaccurrate sputum cytodiagnosis significantly affected the outcome, as there is not even a slight trend, in favour of the active treatment arm.

Heimburger's study is also difficult to compare with the present report due to different proportion of subjects with more severe grades of atypia. Only eight of 73 (10.9%) subjects entered in Heimburger's trial had moderate or severe atypia while in our study 110 of 150 (73%) had moderate or severe atypia at the time of study entry. Although the drugs studied differed, this lends some further support to the hypothesis that an effective chemopreventive intervention may only affect earlier stages of squamous metaplasia but this remains to be tested in subsequent trials.

Zelen (1988) has pointed out the detrimental effect of poor compliance on the statistical efficiency of chemoprevention studies. At the outset of our study we determined to examine several aspects of compliance at the halfway point of the trial and to make an appropriate adjustment in sample size if necessary. The results of these observations have been previously published in detail (Browman *et al.*, 1989; Arnold *et al.*, 1990). In brief, 88% follow-up visits occurred on schedule with only nine missed visits of a possible total of

380. Of 456 possible sputum samples expected to be returned 443 (97.1%) were actually returned on time. Serum sampling for etretinate levels and pill counts suggested that poor compliance would not be an important issue however a small upwards adjustment of sample size was made at this point. On completion of the study the updated results show that compliance remained high as monitored by pill counts and serum sampling. Compliance was higher than in a recently published chemoprevention study by Greenberg *et al.* (Greenberg *et al.*, 1990) which tested beta-carotene to try and prevent recurrence of basal-cell and squamous cell cancers of the skin. Nevertheless previous studies have shown that pill counts, used alone, to monitor compliance, have been unreliable when checked by biochemical monitoring for drug or metabolite (Bergman & Werner, 1963). This was not our experience as the information obtained from pill counts has been strongly supported by the measurement of etretinate levels. Due to the prolonged half-life of etretinate, it is not possible to assess whether subjects omitted to take pills but the stable levels of etretinate, with each visit, certainly suggest that this was not a significant problem. Thus the proportion of subjects who were non-compliant and the low degree of contamination noted in the placebo arm are unlikely to have significantly affected the overall outcome.

A formal run-in period, using placbeo, to exclude non compliant subjects was not part of our study design, however, those less motivated were unlikely to complete the stages leading to eventual study entry (Arnold *et al.*, 1989). In addition, close attention to detail and a considerable effort to accommodate to the varying schedules of participants contributed to achieving a very high degree of subject compliance (Haynes, 1979).

Toxicity, in this study, was very mild. Furthermore, the observation that the number of subjects requiring dose reductions for toxicity and the number of subjects dropping out were equally distributed between treatment and control arms suggests that the blinding of both subjects and study personnel was effective. The dose reductions, which were required, appear to reflect more an apparent true placebo effect rather than significant toxicity. The dose of etretinate we chose, 25 mg orally daily, was the same as that used by Mathé *et al.*, however the lack of major toxicity may indicate that this dose was too low to effect a change on sputum atypia. The issue of dose intensity of retinoid chemoprevention has been discussed by Band *et al.* (1989). In experimental systems, pharmacologic doses of retinoids have been needed to inhibit tumour development (Crocker & Sanders, 1970). Etretinate is used primarily to treat psoriasis in doses up to 100 mg daily. At this higher dose, side-effects from etretinate can be significant. If a strategy using chemoprevention is to ultimately impact on lung cancer incidence it is very unlikely that any but minor side-effects would be acceptable to an essentially healthy population of smokers. Nevertheless, it is feasible that higher doses of etretinate, than used in this study, may have an impact on sputum atypia, but this remains to be tested.

The use of pharmacological interventions to prevent cancer in subjects known to be at high risk is still experimental (Meyskens, 1990; Boone *et al.*, 1990) and some negative studies are likely. However, investigators have reported preliminary encouraging results from carefully designed controlled trials. Heimburger *et al.* (1988) have demonstrated the efficacy of vitamin $B_{12}$ and folate in a study very similar to the present one. In a series of studies, Hong *et al.* (Hong & Doos, 1985; Hong *et al.*, 1986) have demonstrated that the retinoid 13-*cis*-retinoic acid (isotretinoin) is active in reversing the premalignant condition of leukoplakia and more recently have demonstrated that the drug can prevent the occurrence of second primary malignancies in patients treated for primary tumours of the head and neck (Hong *et al.*, 1990). In a non randomised comparison, etretinate was equally effective as 13-*cis*-retinoic acid against leukoplakia (Koch, 1978). Clearly retinoids, including etretinate, can be effective in reversing some of the carcinogenic changes related to smoking. Hong cites the observation that field concerisation exposes wide areas of epithelial surface to carcinogenic insult. Effective pharmacological agents thus have the potential to reverse or delay development of cancers of several related sites and the continued search for effective agents remains important.

There are several large randomised trials using retinoids or beta-carotene attempting to reduce cancer incidence in volunteer smokers. These studies require enormous resources and will take many years to complete. Our study was an attempt to obtain further biological information on the short-term impact of a retinoid on the intermediate biological endpoint of sputum atypia. The negative result should not discourage other investigators from carrying out similar studies using intermediate endpoints, however, changes in sputum cytology alone may not be sensitive or precise enough. Other studies, presently in progress, or being planned, use bronchial biopsies and squamous cell differentiation markers, such as transglutaminase type I, involucrin and the high molecular weight KI keratin as study endpoints and may provide more useful information in the future (Lippman *et al.*, 1990).

### References

ARNOLD, A.M., JOHNSTONE, B., STOSKOPF, B., SKINGLEY, P. & BROWMAN, G. (1989). Recruitment for an efficacy study in chemoprevention - The Concerned Smoker Study. *Prev. Med.*, **18**, 700-710.

ARNOLD, A.M., BROWMAN, G.P., JOHNSTONE, B., SKINGLEY, P., BOOKER, L. & LEVINE, M.N. (1990). Chemoprevention for lung cancer - evidence for a high degree of compliance. *Cancer Detect Prev.*, **14**, 521-525.

AUERBACH, O., PETRICK, G. & STOUT, A.P. (1956). The anatomical approach to the study of smoking and bronchogenic carcinoma. *Cancer*, **9**, 76-83.

BAND, P., DESCHAMPS, M. & ISRAEL, L. (1989). Retinoid chemoprevention timing and dose intensity. *Cancer Investigation*, **7**, 205-201.

BERGMAN, A.B. & WERNER, R.J. (1963). Failure of children to receive penicillin by mouth. *N. Engl. J. Med.*, **268**, 1334-1338.

BOONE, C.W., KELLOFF, G.J. & MALONE, W.E. (1990). Identification of candidate cancer chemopreventive agents and their evaluation in animal models and human clinical trials: a review. *Cancer Res.*, **50**, 2-9.

BROWMAN, G.P., ARNOLD, A., D'SOUZA, T., TURNER-SMITH, L., CAYCO, R. & JOHNSTONE, B. (1990). Use of screening phase data to evaluate observer variation of sputum cytodiagnosis as an outcome measure in a chemoprevention trial. *Cancer Res.*, **50**, 1216-1219.

BROWMAN, G.P., ARNOLD, A., BOOKER, L., JOHNSTONE, B., SKINGLEY, P. & LEVINE, M.N. (1989). Etretinate blood levels in monitoring of compliance and contamination in a chemoprevention trial. *J. Natl Cancer Inst.*, **81**, 795-798.

CROCKER, J. & SANDERS, L. (1970). Influence of vitamin A and 3,7-dimethyl-2,6-octadienal (citral) on the effect of benzo(a)pyrene on the hamster trachea in organ culture. *Cancer Res.*, **30**, 1312-1318.

GREENBERG, E.R., BARON, J.A., STUKEL, T.A., STEVENS, M.M., MANDEL, J.S., SPENCER, S.K., ELIAS, P.M., LOWE, N., NIERENBERG, D.W., BAYRD, G., VANCE, J.C., FREEMAN, D.H., CLENDENNING, W.E., KWAN, T. AND THE SKIN CANCER PREVENTION STUDY GROUP (1990). A Clinical trial of beta-carotene to prevent basal-cell and squamous-cell cancers of the skin. *N. Engl. J. Med.*, **323**, 789-795.

HAYNES, R.B. (1979). Determinants of compliance: the disease and the mechanics of treatment. In *Compliance in Health Care*. Haynes, R.B., Taylor, D.W. & Sackett, D.L. (eds). pp. 49-62. John Hopkins University Press: Baltimore.

HEIMBURGER, D., ALEXANDER, B., BIRCH, R., BUTTERWORTH, C., BAILEY, W. & KRUMDIECK, C. (1988). Improvement in bronchial squamous metaplasia in smokers treated with folate and vitamin B12. *JAMA.*, **259**, 1525-1530.

HONG, W.K., LIPPMAN, S.M., ITRI, L.M., KARP, D.D., LEE, J.S., BYERS, R.M., SCHANTZ, S.P., KRAMER, A.M., LOTAN, R., PETERS, L.J., DIMERY, I.W., BROWN, B.W., GOEPFERT, H. (1990). Prevention of second primary tumours with isotretinoin in squamous-cell carcinoma of the head and neck. *N. Engl. J. Med.*, **323**, 795–801.

HONG, W.K. & BROMER, R. (1983). Chemotherapy in head and neck cancer. *N. Engl. J. Med.*, **308**, 75–79.

HONG, W.K., ENDICOTT, J., ITRI, L.M., DOOS, W., BATASAKIS, J.G., BELL, R., FOFONOFF, S., BYERS, R., ATKINSON, E.N., VAUGHAN, C., TOTH, B.D., KRAMER, A., DIMERY, I.W., SKIPPER, P. & STRONG, S. (1986). 13-cis-retinoic acid in the treatment of oral Leukoplakia. *N. Engl. J. Med.*, **315**, 1501–1505.

KOCH, H.F. (1978). Biochemical treatment of precancerous oral lesions: the effectiveness of various analogs of retinoic acid. *J. Maxillofac Surg.*, **6**, 59–63.

LIPPMAN, S., KESSLER, J. & MEYSKENS, F. (1987). Retinoids as preventive and therapeutic anticancer agents (part I). *Cancer Treat Rep.*, **71**, 391–405.

LIPPMAN, S., LEE, J., LOTAN, R., HITTELMAN, W., WARGOVICH, M. & HONG, W. (1990). Biomarkers as intermediate endpoints in chemoprevention trials. *J. Natl Cancer Inst.*, **80**, 555–560.

MATHÉ, G., GOUVEIA, J., HERCEND, T., GROS, F., DORVAL, T., HAZON, J., MISSET, J.L., SCHWARZENBERG, L., RIBAUD, P., LEMAIGRE, G., SANTELLI, G., REIZENSTEIN, P., HOMASSON, J.P., GAILLARD, J.P., ANGEBAULT, M., BONNIOT, J.P., LEDETENTE, A., MARSAC, J., PARROT, R. & GAGET, H. (1982). Correlation between precancerous bronchial metaplasia and cigarette consumption, and preliminary results of retinoid treatment. *Cancer Detection & Prevention*, **5**, 461–466.

MCCLEAN, S.W., RUDDEL, M.E. & GROSS, E.G. (1982). Liquid chromatographic assay for retinol (vitamin A) and retinol analogues in therapeutic trials. *Clin. Chem.*, **28**, 693–696.

MEYSKENS, F. (1990). Coming of age – the chemoprevention of cancer. *New Engl. J. Med.*, **323**, 825–827.

PAPANICOLAOU, G.N. (1954). *Atlas of Exfoliative Cytology*. Harvard University Press: Cambridge, MA.

SACCOMANNO, G., ARCHER, V.E. AUERBACH, O., SAUNDERS, R.P. & BRENNAN, L.M. (1974). Development of carcinoma of the lung as reflected in exfoliated cells. *Cancer*, **33**, 256–270.

SACCOMANNO, G., SAUNDERS, R.P., ARCHER, V.E., AUERBACH, O., KUSCHNER, M. & BECKLER, P.A. (1965). Cancer of the lung: the cytology of sputum prior to the development of carcinoma. *Acta Cytol.*, **9**, 413–423.

SACCOMANNO, G., MORAN, P., SCHMIDT, R., HARTSHORN, D., BRIAN, D., DREHAR, W. & SOWADA, B. (1982). Effects of 13-cis retinoids on premalignant and malignant cells of lung origin. *Acta Cytol.*, **26**, 79–85.

SAFFIOTTI, U., MONTESANO, R., SELLAKUMAR, A.R. & BORG, S.A. (1967). Experimental cancer of the lung. Inhibition by vitamin A of the induction of tracheobronchial squamous metaplasia and squamous cell tumors. *Cancer*, **20**, 857–864.

SPORN, M.B., ROBERTS, A.B., ROCHE, N.S., KAGECHIKA, H. & SHUDO, K. (1986). Mechanism of action of retinoids. *J. Am. Acad. Dermatol.*, **15**, 756–764.

WILLETT, W.C., POLK, B.F., UNDERWOOD, B.A., STAMPFER, M.J., PRESSEL, S., ROSNER, B., TAYLOR, J.O., SCHNEIDER, K. & HAMES, C. (1984). Relation of serum vitamins A and E and carotenoids to the risk of cancer. *N. Engl. J. Med.*, **310**, 430–434.

ZELEN, M. (1988). Are primary cancer prevention trials feasible? *J. Natl Cancer Inst.*, **80**, 1442–1444.